# The Synergistic Effect of Everolimus and Chloroquine on Endothelial Cell Number Reduction Is Paralleled by Increased Apoptosis and Reduced Autophagy Occurrence

**DOI:** 10.1371/journal.pone.0079658

**Published:** 2013-11-11

**Authors:** Anna Grimaldi, Maria Luisa Balestrieri, Nunzia D'Onofrio, Gilda Di Domenico, Cosimo Nocera, Monica Lamberti, Giuseppe Tonini, Alice Zoccoli, Daniele Santini, Michele Caraglia, Francesco Pantano

**Affiliations:** 1 Department of Biochemistry, Biophysics and General Pathology, Second University of Naples, Naples, Italy; 2 Blood Transfusion Center, “S. Giovanni Bosco” of Naples, Naples, Italy; 3 Department of Experimental Medicine, Section of Igiene, Medicina del lavoro e Medicina Legale, Second University of Naples, Naples, Italy; 4 Campus Bio-Medico University, Translational Oncology PRABB, Rome, Italy; Istituto dei tumori Fondazione Pascale, Italy

## Abstract

Endothelial Progenitor Cells (EPCs), a minor subpopulation of the mononuclear cell fraction in peripheral blood, play a critical role in cancer development as they contribute to angiogenesis-mediated pathological neovascularization. In response to tumor cytokines, including VEGF, EPCs mobilize from the bone marrow into the peripheral circulation and move to the tumor bed where they incorporate into sprouting neovessels. In the present study, we evaluated the effects of everolimus (Afinitor, Novartis), a rapamycin analogue, alone or in combination with chloroquine, a 4-alkylamino substituted quinoline family member, one of the autophagy inhibitors, on EPCs biological functions. We found that either everolimus or chloroquine induce growth inhibition on EPCs in a dose-dependent manner after 72 h from the beginning of incubation. The combined administration of the two drugs to EPC was synergistic in inducing growth inhibition; in details, the maximal pharmacological synergism between everolimus and chloroquine in inducing growth inhibition on EPCs cells was recorded when chloroquine was administered 24 h before everolimus. Moreover, we have studied the mechanisms of cell death induced by the two agents alone or in combination on EPCs and we have found that the synergistic effect of combination on EPC growth inhibition was paralleled by increased apoptosis induction and reduced autophagy. These effects occurred together with biochemical features that are typical of reduced autophagic death such as increased co-immunoprecipitation between Beclin 1 and Bcl-2. Chloroquine antagonized the inhibition of the activity of Akt→4EBP1 axis mediated by everolimus and at the same time it blocked the feed-back activation of Erk-1/2 induced by RAD in EPCs. These data suggest a new strategy in order to block angiogenesis in tumours in which this process plays a key role in both the sustainment and spreading of cancer cells.

## Introduction

EPCs are classically defined as precursor cells recruited from the bone marrow (BM) andvidentified by specific cell surface markers such as CD34, CD133, and Vascular Endothelial Growth Factor Receptor-2 (VEGFR-2) [1]. This progenitor cell population, which incorporate into nascent vessels at angiogenic sites, proliferate and differentiate into the endothelial cells (EC), thus, constituting the blood vessel, have attractively potential prognostic and therapeutic applications to malignant tumors [2], [3]. Indeed, their number and functional changes can be used as a biomarker of the response of cancer patients to anti-tumor therapy and predict the prognosis and recurrence [4], [5]. Neovascularization processes associated with tumour growth are in part supported by recruitment of BM–derived Endothelial Progenitor Cells (EPCs) and their functional incorporation into the new vasculatures. EPCs are mobilized from the bone marrow and respond to growth factors and cytokines that are released into the circulation by injured endothelium and tissues or tumour tissues [6], [7]. The BM compartment activation results in the EPCs mobilization and recruitment to the tumour bed where they take part to the formation of neovasculature and tumor growth in adults [6], [7].

The formation of blood vessels occurs by two mechanisms: vasculogenesis and angiogenesis. Vasculogenesis is the process where blood vessels are formed de novo by in situ differentiation of angioblasts into mature EC [6], [7]. In contrast, angiogenesis occurs both during embryonic development and postnatal life and is defined as a process that gives rise to new blood vessels by proliferation and migration of pre-existing differentiated EC [6], [7]. It has been also demonstrated the existence of additional angiogenic and vasculogenic mechanisms associated with tumor growth, such as intussusceptive angiogenesis, vessel co-option, vasculogenic mimicry, lymphangiogenesis, and the recruitment EPCs [8]. In most cases, these mechanisms take place concomitantly and are the potential targets for novel antiangiogenic/antitumor therapeutic strategies [8].

EPCs facilitate the initial establishment of tumour endothelium, control tumor growth and metastasis transition [9]–[11]. Notably, these cells can determine the sensitivity of a tumor to chemotherapeutics [12], [13]. Clinical studies report that decline of EPC numbers correlates with response to chemotherapy whereas the presence of circulating EPC positively correlates with advanced invasive stages [14], [15], suggesting that EPC numbers can be a marker of both cancer progression and chemotherapeutic efficacy.

Everolimus, a rapamycin analogue, is an oral mammalian target of rapamycin (mTOR) inhibitor which belongs to the PI3K related family of protein kinases and is activated by phosphorylation at serine 2448 (S2448) [16], [17]. mTOR is a key effector in the PI3K/Akt/mTOR pathway and plays a critical role in regulating cell proliferation, survival, and angiogenesis [18]. mTOR works as part of two distinct multimeric complexes, known as MTORC1 and MTORC2. MTORC1 phosphorylates S6K1 and eIF-4E-binding protein 1 (4E-BP1) leading to protein synthesis. MTORC2 phosphorylates AKT in response to growth factors. As negative feedback, MTORC1 inhibits AKT by suppressing upstream pathways. The complexity of MTOR system implies contradictory functions both as tumor promoting and suppressor factors [19]. Furthermore, the PI3K/Akt/mTOR pathway is involved in the control of autophagy, an ubiquitous and evolutionarily conserved process that degrades cytosolic components via the lysosomes and allows cells to survive to various forms of stress [20]. There is increasing evidence that PI3K/Akt/mTOR inhibitors initiate autophagy as a survival program that may interfere with their antitumor activity. Consequently, inhibition of autophagy was used as a strategy to enhance the efficacy of PI3K/Akt/mTOR inhibitors in different cancers [21]–[23]. Everolimus has been approved for second-line therapy of patients with renal cell carcinoma after failure of treatment with sunitinib and for the treatment of papillary renal carcinoma, pancreatic neuroendocrine tumor, some types of breast cancer, and subependymal giant cell astrocytoma associated with tuberous sclerosis [24]–[28].

Interestingly, rapamycin and its analogs, temsirolimus (CCI-779) and everolimus have been shown to have suppressive effects on the tumor cell compartment as well as on tumor angiogenesis [29]–[31]. Increasing experimental and clinical evidence may establish mTOR inhibition as a promising rationale for targeting human malignancy.

Autophagy is known to limit tumor cell growth, reduce mutagenesis or other damage caused by reactive oxygen species [32]–[34]. Alternatively, autophagy may kill developing tumor cells. Stimulation of autophagy suppresses cancer development but may promote tumor growth and survival under conditions of nutrient deprivation in poorly angiogenic tumors. Autophagy may also protect tumor cells from undergoing apoptosis in response to treatment with anticancer agents. These evidences suggest that, depending on the context, autophagy can both stimulate and prevent cancer. To date, there are several autophagy inhibitors are used to study the role of autophagy. Chloroquine, a 4-alkylamino substituted quinoline family member, is one of the autophagy inhibitors. It is lysosomotropic and inhibits the fusion of autophagosomes and lysosomes. Chloroquine is widely used as an anti-malarial, and also as an anti-inflammatory in the treatment of rheumatoid arthritis and lupus erythematosus. Recently, attention has focused on its potential as an anti-cancer agent and chemotherapy sensitizer [35], [36]. Although the precise basis of the anti-cancer effects of chloroquine is still under investigation, inhibition of autophagy most probably plays a part in anti-tumoral effect.

In the present study, we evaluated the effects of everolimus (RAD) alone or in combination with Chloroquine (CLC) on EPCs number and investigated possible mechanisms by which these two agents alone or in combination affect EPCs viability. Moreover, we have designed a new strategy in order to block angiogenesis in tumours in which such a process plays an important role in both the sustainment and spreading as in the case of renal cancer.

## Materials and Methods

### EPC isolation and characterization

EPCs were isolated from total peripheral blood mononuclear cells (PBMCs). Briefly, PBMCs of healthy human donors (who signed an informed consent for the use of the derived biological samples after approval of the protocol by the Institutional Review Board of the Department of Biochemistry, Biophysics and General Pathology of the Second University of Naples) were isolated by density gradient centrifugation of 15 ml of leukocyte-rich buffy coat on Histopaque-10771 (1.077 g/ml; Sigma- Aldrich) (400 g for 40 min at 4°C) according to the manufacturer's protocol. After centrifugation the interface cells were carefully removed and transferred to a new conical tube. Cells were washed twice with PBS, centrifuged at 300 g for 10 min at 4°C and then suspended in 9 ml of H_2_O, 3 ml KCl 0.6 M to a final volume of 50 ml of PBS (1×). Isolated PBMCs (∼200×10^6^ cells) were plated on fibronectin-coated culture dishes (5×10^6^ cells/ml medium) and maintained in endothelial basal medium- 2 (EBM-2) supplemented with 0.1% rhEGF, 0.1% VEGF, 0.4% rhFGF-B, 0.1% R3–IGF-1, 0.1% ascorbic acid, 0.1% heparin, 0.1% GA-1000 (gentamycin), 0.04% hydrocortisone, and 2% FBS (EGM-2 Singlequots, Lonza). In order to confirm endothelial phenotype (CD34+/KDR+), the adherent EPC were trypsinized for 2 min, then the reaction was stopped with EBM-2 complete medium. Flow cytometry analysis (FACS) was performed by incubating samples with directly conjugated mouse monoclonal antibodies to human CD34-FITC (Macs) and to anti-h VEGFR2/KDR-PE (R&D System, USA). Quantitative fluorescence analysis was performed with a FACS-CANTO instrument (BD Biosciences).

### Drug combination studies

For drug combination studies between RAD (Afinitor, Novartis) and CLC (Sigma, Aldrich) on EPCs, the isolated PBMCs cells were seeded in 6-multiwell plates at the density of 5×10^6^ cells/ml medium. After 24 h at 37°C with 5% CO_2_ in a humidified atmosphere, the cells were treated with different concentrations of RAD and CLC. In details, we have assessed the effects of different concentrations of the two drugs (12.5, 25 and 50 µM for RAD and 20, 40 and 80 µM for CLC) on the growth of EPCs. Subsequently, we have evaluated the effects of different sequences of administration of the combinations of the two drugs: i) RAD for 48 h and CLC for 72 h (CLC → RAD), **ii**) RAD for 72 h and CLC for 48 h (RAD→ CLC), iii) in co-administration of RAD and CLC (RAD/CLC) for 72 h. At the end of the treatments FACS computed counting of (CD34+/KDR+) was performed as described.

### Apoptosis Assay

Annexin V-FITC (fluorescein isothiocyanate) was used in conjunction with a vital dye, Propidium Iodide (PI), to distinguish apoptotic (Annexin V-FITC positive, PI negative) from necrotic (Annexin V-FITC positive, propidium iodide positive) cells. Annexin-V–FITC binds to phosphatidylserine molecules, which are translocated from the inner to the outer leaflet of the plasma membrane during the early stages of apoptosis. Briefly, the isolated PBMCs cells were seeded in 6-multiwell plates at the density of 5×10^6^ cells/ml medium and then incubated 24 h in humidified atmosphere at 37°C. The day after cells were treated with either the single agents or the synergistic sequence. After the treatment the cells were collected and centrifuged for 5 min at 1,300 rpm. Pellet was washed in PBS, cells were incubated with Annexin-V-FITC (MedSystems Diagnostics, Vienna, Austria) and PI (Sigma, St. Louis, MO, USA) in a binding buffer (10 mM HEPES, pH 7.4, 150 mM NaCl, 5 mM KCl, 1 mM MgCl_2_, 2.5 mM CaCl_2_) for 10 min at room temperature, washed and resuspended in the same buffer as described by the manufacturer. Analysis of apoptotic cells was performed by flow cytometry (FACScan, Becton Dickinson). For each sample, 1×10^4^ events were acquired. Analysis was carried out by triplicate determination on at least three separate experiments.

### Autophagy assay

To quantify the induction of the autophagic process in EPC treated with drugs, the autofluorescent agent monodansylcadaverine (MDC), a selective marker for autophagic vacuoles (AVOs) and especially autolysosomes, staining was performed, after the treatment with CLC (20 µM) → RAD (6.5 µM) for 72 h. Following the treatments, cells were incubated with 50 µM MDC in PBS at 37°C for 15 min. After incubation, cells were washed with PBS, trypsinized, and immediately analyzed by flow cytometry. All fluorescences were analyzed with a FACScalibur flow cytometer (Becton Dickinson). The fluorescent emissions were collected through a 530 nm band pass filter (FL1 channel). At least 10,000 events were acquired in log mode. For the quantitative evaluation of MDC, CellQuest software (Becton Dickinson) was used to calculate mean fluorescence intensities (MFIs). The MFIs were calculated by the formula (MFItreated/MFIcontrol), where MFItreated is the fluorescence intensity of cells treated with the various compounds and MFIcontrol is the fluorescence reported in the figures are the means ± S.D.s from three independent experiments.

### Western blot analysis

EPCs were grown for 72 h with or without RAD and/or CLC in the previously described experimental conditions. For cell extract preparation, cells were washed twice with ice-cold PBS/BSA, scraped, and lysed for 30 min at 4°C in lysis buffer (50 mM Tris, pH 7.4, 150 mM sodium chloride, 1% Nonidet P-40, 1 mM EDTA, 1 mM sodium orthovanate, 1 mM sodium fluoride, 1 µg/ml leupeptin, 1 µg/ml aprotinin, 1 µg/ml pepstatin A, 1 mM phenylmethylsulfonyl fluoride (PMSF)). Lysates were spinned at 10000×g for 10 min and supernatants were collected. Protein concentration was determined by Lowry method and compared with bovine serum albumin standard curve. Approximately, 40 µg of protein extract were separated by 12.5% SDS-PAGE as described [21], [32], [37], [38]. The gel was transblotted by Trans blot turbo (BIORAD) on a nitro-cellulose membrane and reacted with the different MAbs: anti-AKT and anti-pAKT (Cell Signaling Tecnology Inc, Beverly, MA, USA), anti-Beclin1 (Cell Signaling Tecnology Inc, Beverly, MA, USA), anti Bcl2 (Santa Cruz Biotechnology, inc.), anti-p-4EBP1 (Cell Signaling Tecnology Inc, Beverly, MA, USA), anti-MAPK p42/44 and anti-p-MAPK p42/44 (Cell Signaling Tecnology Inc, Beverly, MA, USA), anti-pP38 (Thr180/Tyr182) (3D7) (Cell Signaling Tecnology Inc, Beverly, MA, USA). After incubation with secondary antibodies, the signal was detected using enhanced chemoluminescence detection reagents (SuperSignal West Pico, Pierce) and exposed to X-ray film. The bands derived from western blotting were scanned with a laser scanner (Epson 1260) and their intensities were quantified with Image J software (NIH). Membranes were normalized with a polyclonal antibody against tubulin protein (GTU-88) (Sigma Aldrich). The error bars shown in the histograms represent the standard deviation from the mean of different densitometric scanning in at least three different experiments.

### Immunoprecipitation

Protein lysates (120 µg) were incubated with primary antibody (anti-Beclin1, Cell Signaling and anti-Bcl2, Enzo Life) overnight at 4°C, with gentle rocking. Immune complexes were collected with 20 µl of protein A-agarose and incubated for 16 h at 4°C. The protein A-agarose/immune complex was washed twice with cold PBS, resuspended in 20 µl of SDS-loading buffer, heated to 95°C for 5 min and used for western blotting analysis using either anti-bcl2 or anti-beclin 1 antibodies.

### Statistical analysis

Data are given as mean ± DS. Differences were assessed by *t* test and *p*<0.05 was considered to be significant.

## Results

### RAD and CLC reduce EPC number

**Figure 1:**
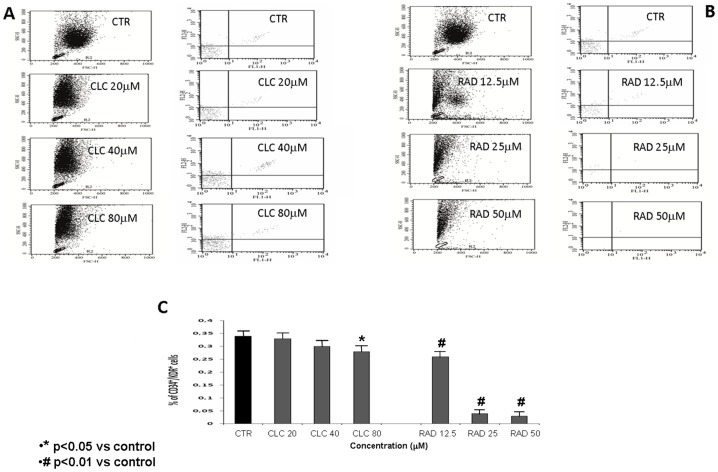
FACS analysis of samples of PBMNC derived from human healthy donors (see “Materials and Methods”) labelled with mouse monoclonal antibodies conjugated with fluorophores (CD34-FITC and KDR-PE). Quantitative fluorescence analysis was performed with a FACS-CANTO instrument (BD Biosciences). Cells positive for both CD34-FITC and KDR-PE were considered as EPC. Isolated PBMCs incubated with CLC (20, 40 and 80 µM) (Fig.1 A) or RAD (12.5, 25 and 50 µM) (Fig.1 B) for 72 h in complete media at 37 C. Control cells were also cultured in complete media for 72 h at 37 C. Fig. 1 C shows the CD34+/KDR+ cells after treatment with CLC (20, 40 and 80 µM (p<0.05)) or RAD (12.5, 25 and 50 µM) (p<0.01) expressed as % of control. The figure is representative of three different experiments that always gave similar results.

RAD and CLC reduced EPCs number in a dose-dependent manner after 72 h from the beginning of incubation (Figure 1). Number of CD34+/KDR+ cells was significantly reduced after treatment with 12.5 µM, 25 µM and 50 µM RAD (p<0.01) (Figure 1B), while reduction following CLC treatment was less pronounced (p<0.05) (Figure 1A). Figure 1C shows the % of CD34^+^/KDR^+^cells after treatment with CLC (20, 40 and 80 µM) and RAD (12.5, 25 and 50 µM), as percentage of the untreated EPCs.

### CLC synergizes with CLC in the reduction of EPC number

**Figure 2:**
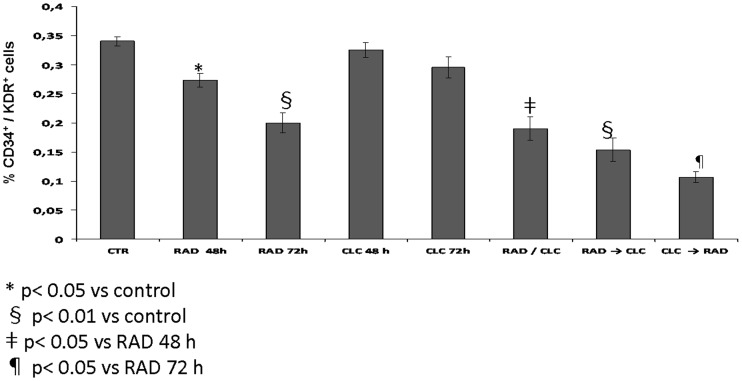
Evaluation of CLC and RAD effect on EPC number. CLC20 µM and RAD 12.5 µM were added in different sequences of administration (untreated cells, CTR; CLC added for 72 h, CLC 72 h; CLC added for 48 h, CLC 48 h; RAD added for 72 h, RAD 72 h; RAD added for 48 h, RAD 48 h; CLC added for 72 h and RAD for the last 48 h, CLC → RAD; RAD added for 72 h and CLC for the last 48 h, RAD → CLC; CLC and RAD added both for 72 h, CLC/RAD for 72 h). Control cells were cultured in complete media for 72 h at 37°C. The figure shows the % of CD34+/KDR+ cells after treatment with CLC and RAD, alone, and in sequence, compared to the control. The experiments were repeated three times and the values are the mean ± SD of the three independent experiments.

**Figure 3:**
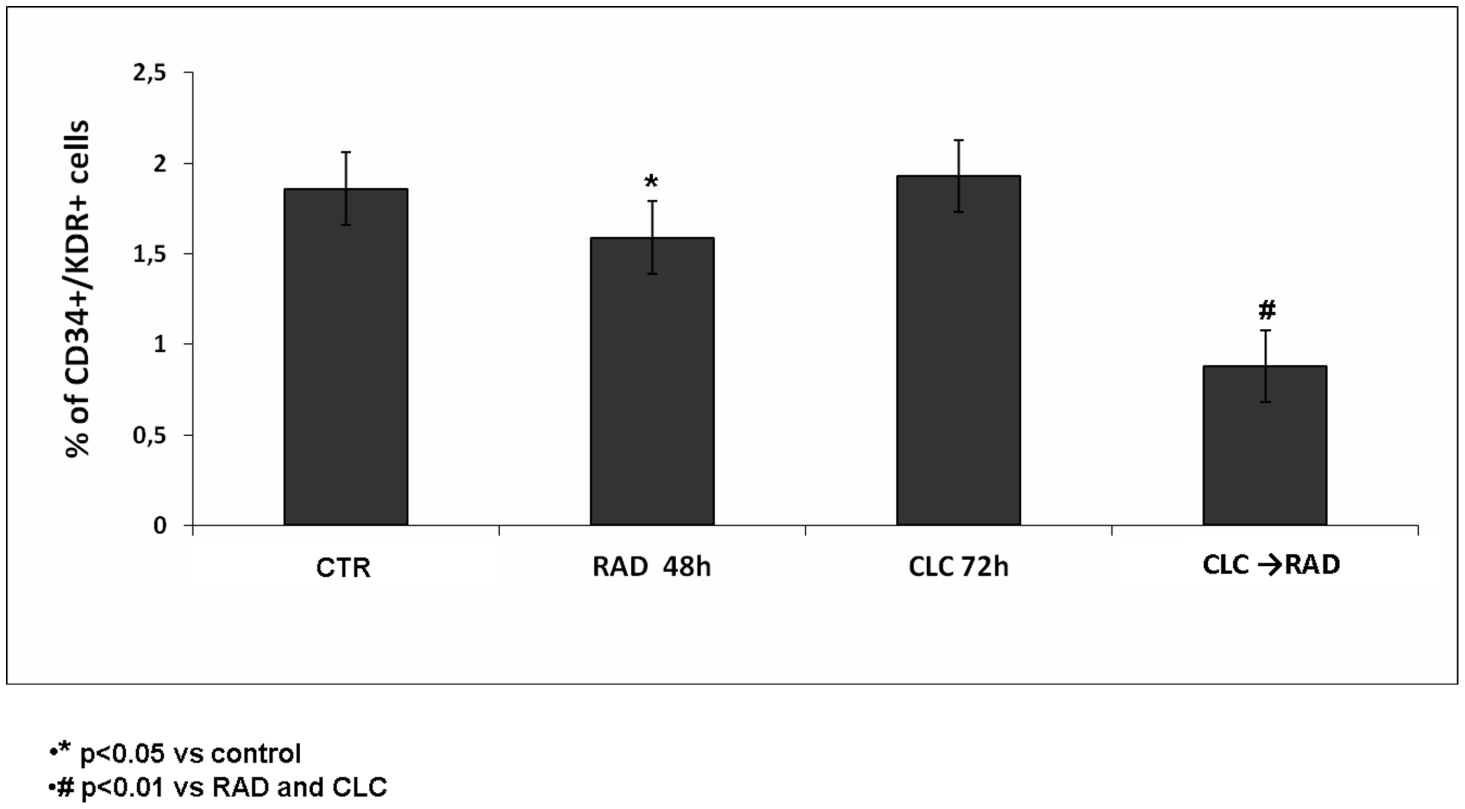
Evaluation of CLC and RAD effect on EPC number. CLC 20 µM and RAD 6.5 µM were added in different sequences of administration (untreated cells, CTR; CLC added for 72 h, CLC 72 h; RAD added for 48 h, RAD 48 h; CLC added for 72 h and RAD for the last 48 h, CLC → RAD). Control cells were cultured in complete media for 72 h at 37°C. The figure shows the % of CD34+/KDR+ cells after treatment with CLC and RAD, alone, and in sequence, compared to the control. The experiments were repeated three times and the values are the mean ± SD of the three independent experiments.

Then, we selected two concentrations of RAD and CLC (12.5 µM RAD and 20 µM CLC) that induced a reduction of cell number of about 20% and 6%, respectively. Subsequently, we evaluated the effects of the two agents alone or in combination in different sequences of administration as previously described in “Materials and Methods” section. Figure 2 shows the % of CD34^+^/KDR^+^ (percentage of the control cells) after treatment with CLC and RAD alone or in sequence. The reduction of EPC number induced by the single agents was of about 20% and 42% for RAD 48 h and 72 h, respectively. On the contrary, The reduction of EPC number induced by CLC was 6% and 12% at 48 h and 72 h, respectively. The reduction of EPC number caused by the combination of the two drugs in the sequences of administration were of about 68% in CLC → RAD, 56 % in RAD → CLC and 45% in CLC+RAD, respectively. On the basis of the data obtained, we decreased the concentration of RAD (from 12.5 µM to 6 µM) in order to reduce the effect of the single agent on the EPC number. Similarly, also in this case we observed a decreased EPC number induced by the CLC → RAD sequence compared to drugs alone (Figure 3). In fact, CLC and RAD alone induced 12% and 5% (p<0.05) number reduction, respectively, while the reduction caused by the combination of the two drugs in the sequences of administration were of about 60 % in CLC → RAD (p<0.01).

### The synergistic effect of the CLC/RAD combination is mediated by apoptosis induction

**Figure 4:**
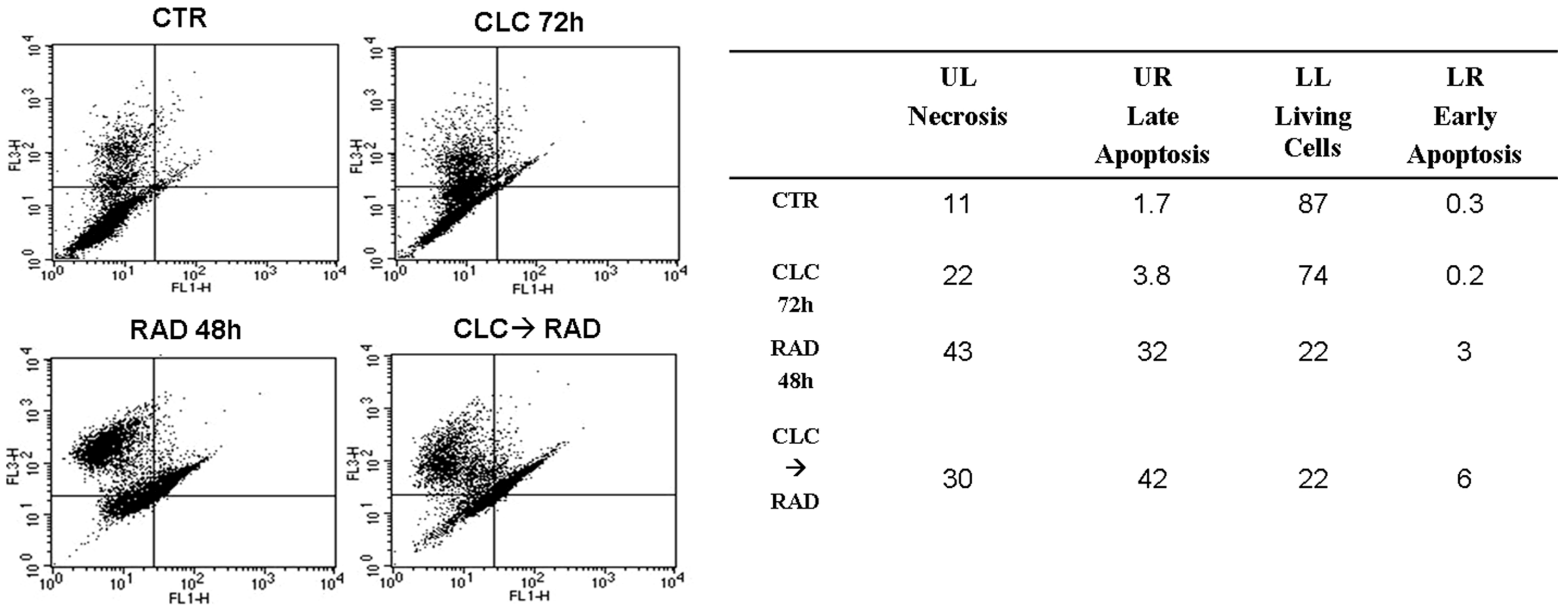
FACS analysis after double labelling EPCs with PI and Annexin V. WPCs were treated with CLC and RAD alone and in sequence, compared to the control. The experiments were performed at least three times and the results were always similar. Insets, % of positive cells. **UL**  =  Upper Left (necrosis); UR  =  Upper Right (late apoptosis); **LL**  =  Lower Left (viable); **LR**  =  Lower Right (early apoptosis). Untreated cells, CTR; CLC added for 72 h, CLC 72 h; RAD added for 48 h, RAD 48 h; CLC added for 72 h and RAD for the last 48 h, CLC → RAD. The figure is representative of three different experiments that always gave similar results. The table show the percentage of cells in the different quadrants.

After the treatment with CLC (20 µM) → RAD (6.5 µM), we have evaluated the induction of apoptosis on EPCs by FACS analysis, after staining with Annexin V-FITC and PI, as described above. As shown in Figure 4, we found a significant increase of apoptotic cells treated with the two drugs in combination, compared to untreated cells or cells treated with the single agents. In details, we found that the treatment with CLC alone for 72 h induced apoptosis in only about 3.8% of EPC population, while the treatment with RAD for 48 h alone induced apoptosis in about 32% (p<0.01) of EPC population compared to 1.7 % of untreated cells. On the other hand, when the cells were treated with the sequence CLC → RAD apoptosis was recorded in about 42% of EPC (p<0.01). On the basis of these findings, it can be assumed that the combination was able to induce synergistic effects on apoptosis occurrence that was paralleled by the synergism on cell growth inhibition.

### CLC/RAD combination reduced autophagy induced by RAD

**Figure 5:**
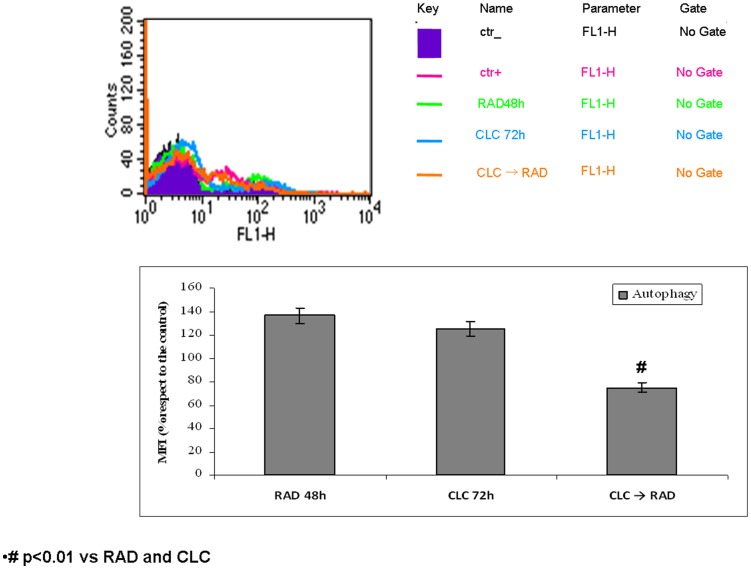
Autophagy evaluation. A) After treatment with CLC and/or RAD alone or in sequence, EPCs were incubated with MDC and analyzed by flow cytometry as described in “Materials and Methods” in order to evaluate the autophagy onset. Untreated cells unexposed to MDC, CTR^-^; untreated cells exposed to MDC, CTR^+^; CLC added for 72 h, CLC 72 h; RAD added for 48 h, RAD 48 h; CLC added for 72 h and RAD for the last 48 h, CLC → RAD. B) Representation of autophagy expressed as percentage of mean fluorescence intensity (MFI) derived by MDC stained EPC cells treated with CLC and/or RAD alone or in sequence. CLC added for 72 h, CLC 72 h; RAD added for 48 h, RAD 48 h; CLC added for 72 h and RAD for the last 48 h, CLC → RAD. The experiments were repeated at least three times and always gave similar results. Bars, SDs.

Moreover, we studied the effects of the two agents alone or in combination on autophagy occurrence, an alternative cell death mechanism that could be involved in the triggering of tumour cell resistance to RAD. Autophagy was evaluated by FACS analysis after staining with autofluorescent molecule associated to early autophagosomes MDC, as described in “Materials and Methods” section. As shown in Figure 5, treatment of EPC with CLC and RAD alone induced a 25 and 36 % increase of MFI, respectively, if compared to untreated cells, while the treatment with the sequence CLC→RAD restored the autophagy levels to those of untreated cells (p<0.01). These findings suggest that when synergistic conditions on both EPCs growth inhibition and apoptosis were recorded autophagy occurrence was almost completely antagonized by the sequential treatment with the two agents.

### Molecular mechanisms of autophagy induction

**Figure 6:**
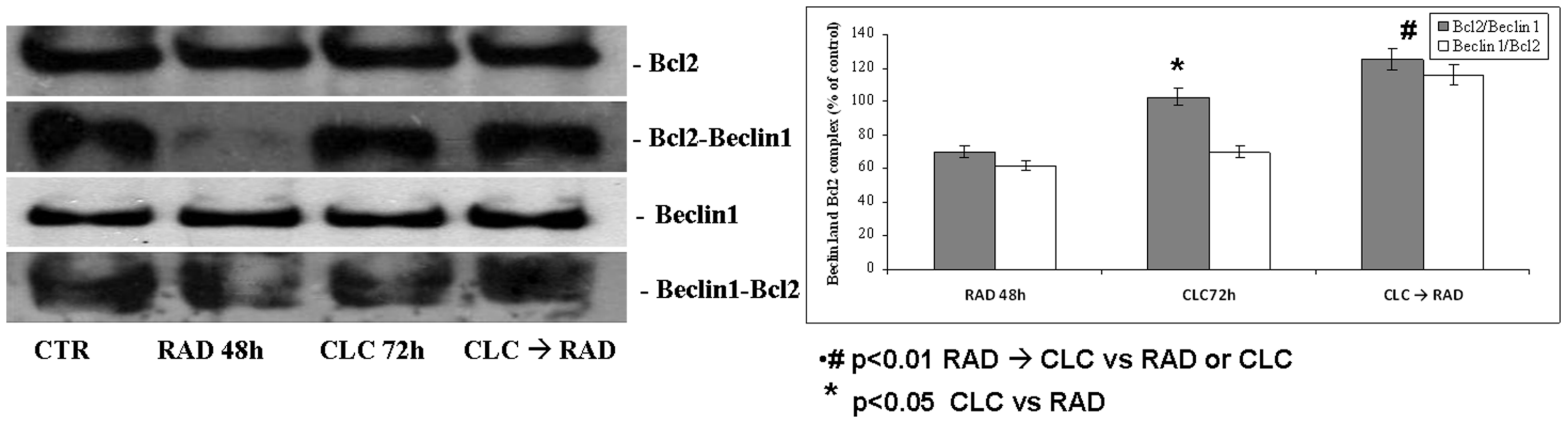
Evaluation of Beclin-1 and Bcl-2 interaction. A) EPCs were treated with CLC and RAD alone or in sequence and thereafter proteins were extracted. Then we performed western blot assay for the expression of the total Bcl2 and Beclin-1 proteins and immunoprecipitation of Bcl-2 and Beclin-1 subsequently blotted with either anti-Beclin-1 or Bcl-2 antibodies, respectively, for the evaluation of Bcl-2/Beclin-1 and Beclin-1/Bcl-2 complex formation as described in “Material and Methods”. Untreated cells, CTR; CLC added for 72 h, CLC 72 h; RAD added for 48 h, RAD 48 h; CLC added for 72 h and RAD for the last 48 h, CLC → RAD. The figure is representative of three different experiments that always gave similar results. B) Representation of the Bcl2/Beclin-1 complexes expressed as the ratio between the relative intensities of the bands associated with the Bcl-2/Beclin-1 and Beclin-1/Bcl-2 complexes versus the bands associated with total Bcl-2 and Beclin-1, respectively. The intensities of the bands were expressed as arbitrary units when compared to those of the untreated cells. The values are the mean of three independent experiments. SDs, standard deviations.

**Figure 7:**
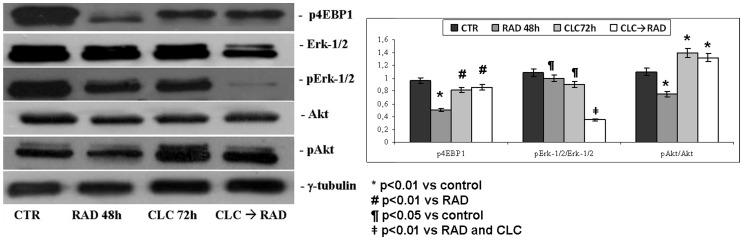
Evaluation of pathways involved in the regulation of proliferation and survival. A) EPCs were treated with RAD and/or CLC alone or in combination. Thereafter, both the activity and the expression of the different proteins were evaluated after blotting with specific antibodies, as described in “Materials and Methods”. Expression of the house-keeping protein γ-tubulin was used as loading control. Untreated cells, CTR; CLC added for 72 h, CLC 72 h; RAD added for 48 h, RAD 48 h; CLC added for 72 h and RAD for the last 48 h, CLC → RAD. The figure is representative of three different experiments that always gave similar results. B) Representation of the intensities of the bands associated to the different proteins normalized for the expression of the total proteins. The intensities of the bands were expressed as arbitrary units when compared to those of the untreated cells. The values are the mean of three independent experiments. SDs, standard deviations.

We next investigated the molecular mechanisms of autophagy studying the interaction between two molecules involved in this process: Beclin-1 and Bcl-2. It is known that Bcl-2, interacting with Beclin-1, inhibits Beclin-1-dependent autophagy [39]. When these proteins were co-immunoprecipitated, we found that combined treatment increased Beclin-1/Bcl-2 complex formation if compared to the two drugs alone (p<0.01) (Figure 6). The expression of total Beclin-1 and Bcl-2 remained unchanged in both the single and sequential treatments. On the other hand, a significant increase of Beclin-1/Bcl-2 complex was recorded in EPCs treated with the two drugs in combination if compared to drugs alone or to untreated EPCs. Another key component that regulates the balance between cell growth and autophagy in response to cellular physiological conditions and environmental stress is mammalian target of rapamycin (mTOR), the target of RAD. The activation of mTOR leads to the phosphorylation of its two major downstream components, p70S6K and eIF4E-binding protein 1 (4EBP1). 4EBP1 is believed to primarily control cap-dependent translation by binding and inactivating eIF4E, an initiation factor, which binds to the mRNA cap structure, thereby mediating the initiation of translation. Phosphorylation of 4EBP1 results in the release of eIF4E and subsequent activation of the eIF4G scaffolding protein. Both PI3 kinase/AKT pathway and mTOR kinase regulate 4EBP1 activity [40]. As shown in Figure 7, we found that the combination between CLC/RAD and treatment with CLC increased 4EBP1 phosphorylation if compared to the RAD (p<0.01) while 4EBP1 phosphorylation was significantly reduced also by RAD alone (p<0.01). Therefore, the antagonism induced by CLC on RAD-mediated autophagy was paralleled by an immobilization of the pro-autophagic protein Beclin 1 mediated by Bcl2 and by a decrease of the phosphorylation of 4EBP1, a target of the anti-autophagic signalling protein mTOR.

### Effects of CLC and RAD on proliferation and survival pathways

Finally, we studied the effects of the drug combination on both the expression and activity of two key molecules involved in the regulation of both cell proliferation and survival processes, MAPK (Erk-1/2) and AKT (Figure 7). In EPCs, CLC and RAD alone caused an about 40% decrease of Erk-1/2 phosphorylation (p<0.05 vs control) while the sequence CLC→RAD induced a stronger reduction of Erk activity (an about 80% decrease) (p<0.01 vs RAD and CLC). On the other hand, no effects on the expression of the proteins were recorded. RAD induced an about 20% decrease of AKT phosphorylation while CLC caused an about 40% increase of Akt phosphorylation that was still augmented in cells treated with the sequential combination (about 20% increase if compared to untreated cells) (p<0.01). Again the treatment of EPCs with all the agents alone or in combination had no effects on the expression of total Akt protein. These data suggest that RAD induced a rebound effect on the activation of Erk-1/2 that was completely antagonized by CLC that, in turn, antagonized the slight decrease induced by RAD on Akt activity.

## Discussion

In the present study, we demonstrated, for the first time to our knowledge, that RAD and CLC reduced EPC number with a synergistic effect. Other major findings of this study are that synergistic effect of the RAD/CLC combination on EPC number is mediated by the induction of apoptosis and reduction of the autophagy induced by RAD treatment. Tumour angiogenesis is prevented by mTOR inhibitors through two mechanisms: i) decreased synthesis and release of angiogenic growth factors (especially VEGF) from the cancer cells and ii) blocked growth by the reduction of vascular cell survival. Indeed, tumor cells release excessive amounts of angiogenic factors, including VEGF, not only because they are deprived of oxygen but also because deregulated oncogenes (HIFs for example) activate their gene transcription [41]–[43]. VEGF and other factors are known to recruit EPCs, explaining why reducing angiogenic factors signalling leads to a reduction of EPC mobilization in cancer patients [44]–[46] and why EPC levels rebound concomitantly with a rise of plasma VEGF and other pro-angiogenic cytokines in patients during drug-free intervals of a pan-VEGF inhibition [47]. Levels of EPCs, correlate with tumor burden [48]–[50], thus, playing also a key role in conferring resistance against anti-angiogenic compounds and some chemotherapy agents [51]. EPCs, unlike malignant cells, are supposed to be genomically stable, except for occasional studies reporting that experimental and human tumors may harbor genetic abnormalities, and would therefore not be able to escape from anti-angiogenic therapy. Therefore, enhancing the direct cytotoxic effect of mTOR inhibitors against the EPCs could result in more sustained and prolonged anti-angiogenic response. In the present study, we evaluated the effects of a clinically available mTOR inhibitor (RAD) on the levels of EPCs isolated from buffy coat of human healthy donors. We found that RAD is, indeed, able to reduce the EPC number, as already reported by other groups [52]. It is noteworthy that mTOR inhibition mediated by rapamycin can induce growth inhibition of EPCs through the triggering of an apoptotic effect [53]. On the other hand, autophagy occurrence was associated to resistance to RAD in some human tumour models [54]. In fact, RAD stimulates autophagy [55], [56], a highly conserved process that entails the degradation of intracellular components through the lysosomal machinery to regenerate metabolites for energy and growth [57], [58]. In many contexts, autophagy promotes cell survival under stressful conditions such as nutrient and growth factor deprivation [59], [60]. Data suggest that mechanism by which combination of mTOR and autophagy blockers mediates an increase in cytotoxic effect is mainly due to a prolonged and sustained oxidative damage. Indeed mTOR inhibition blocks glycolysis metabolism promoting the switch toward a more sustained oxidative phosphorylation leading, in turn, to an increase of oxidative stress linked to ROS generation [61]. In presence of an intact autophagic machinery, the excess of ROS can be controlled by eliminating the mitochondria through mitophagy process thus preserving cell survival. Anyway, following autophagy blockage, the increase of ROS generation cannot be counteracted by mithopagy resulting in a dramatic increase in ROS load, oxidative damage and subsequent cell death [61], [62], [63].

**Figure 8:**
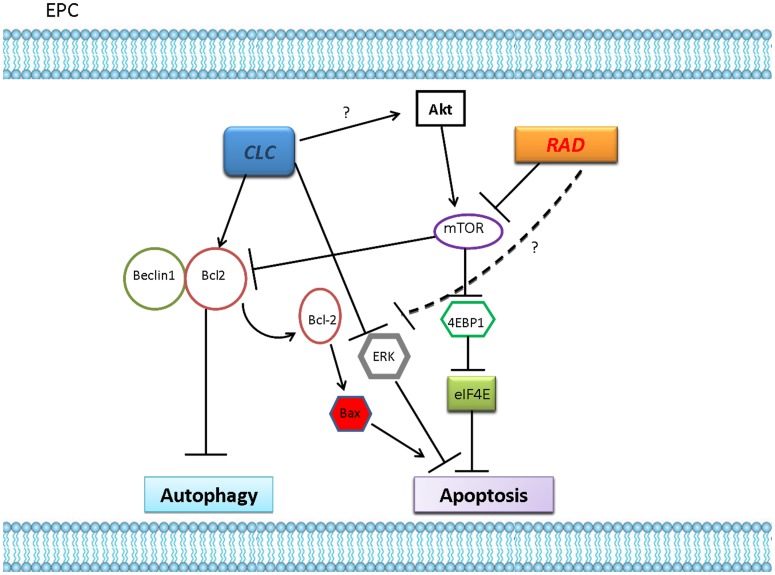
Scheme of the effects induced by RAD/CLC interaction. (Right part) RAD inhibits mTOR and the downstream anti-apoptotic eIF4E-dependent pathway and, at the same time, causes inactivation of the beclin-1/bcl-2 complex formation inhibiting autophagy. Moreover, RAD induces a slight decrease of the activity of the survival enzymes Erk-1 and 2. (Left part) CLC increases the formation of the beclin-1/bcl-2 complexes thus inhibiting autophagy and, at the same time, induces a slight decrease of the Erk-1/2 activity and increases Akt activity. On these bases, the addition of CLC before RAD to EPCs inhibits autophagy occurrence through association of beclin-1 to bcl-2 and has poor effects on apoptosis as it decreases the activity of the pro-survival Erk-1/2, but increases the activity of the anti-apoptotic Akt. The subsequent addition of RAD potentiates the pro-apoptotic effects of CLC as it enhances the inhibition of Erk-1/2 and inhibits, at downstream levels, the Akt-dependent signalling (activated by CLC). In this way, the sequential treatment with the two agents induces a potent switch from autophagy to apoptosis in EPCs.

On the other hand, the understanding of the fine tuning of the signal transduction pathways regulating cell death and controlling the switch from autophagy to apoptosis and *viceversa* is pivotal in the design of new anti-cancer/anti-angiogenic strategies. In this light, our results suggest that RAD alone inhibits mTOR and the downstream anti-apoptotic eIF4E-dependent pathway and, at the same time, causes inactivation of beclin-1/bcl-2 complex formation. Moreover, RAD induces a slight decrease of the activity of the survival enzymes Erk-1 and 2. CLC alone increases the formation of the beclin-1/bcl-2 complexes and, at the same time, induces a slight decrease of the Erk-1/2 activity and increases Akt activity. The addition of CLC before RAD inhibits autophagy occurrence through association of beclin-1 to bcl-2 and has poor effects on apoptosis as it decreases the activity of the pro-survival Erk-1/2, but increases the activity of the anti-apoptotic Akt. The subsequent addition of RAD potentiates the pro-apoptotic effects of CLC strongly inhibiting Erk-1/2 and inactivates, at downstream levels, the Akt-dependent signalling (Figure 8). This mechanism is strongly attractive in the era of selective signal inhibitors in order to design molecularly based anti-cancer strategies. Therefore, in future, double mTor and autophagy inhibition could result in a new therapeutic strategy to selectively kill tumor angiogenic cells.
